# Experimental Study on Aerodynamic Characteristics of Downwind Bionic Tower Wind Turbine

**DOI:** 10.3390/biomimetics9060336

**Published:** 2024-06-02

**Authors:** Junwei Yang, Xin Sun, Hua Yang, Xiangjun Wang

**Affiliations:** 1 Guangling College, Yangzhou University, Yangzhou 225000, China; yangjunwei@yzu.edu.cn; 2 College of Electrical, Energy and Power Engineering, Yangzhou University, Yangzhou 225127, China; sunxinluck@163.com (X.S.); x.j.wang@yzu.edu.cn (X.W.)

**Keywords:** downwind wind turbine, bionic tower, wind tunnel test, aerodynamic characteristics

## Abstract

The vibrissae of harbor seals exhibit a distinct three-dimensional structure compared to circular cylinders, resulting in a wave-shaped configuration that effectively reduces drag and suppresses vortex shedding in the wake. However, this unique cylinder design has not yet been applied to wind power technologies. Therefore, this study applies this concept to the design of downwind wind turbines and employs wind tunnel testing to compare the wake flow characteristics of a single-cylinder model while also investigating the output power and wake performance of the model wind turbine. Herein, we demonstrate that in the single-cylinder test, the bionic case shows reduced turbulence intensity in its wake compared to that observed with the circular cylinder case. The difference in the energy distribution in the frequency domain behind the cylinder was mainly manifested in the near-wake region. Moreover, our findings indicate that differences in power coefficient are predominantly noticeable with high tip speed ratios. Furthermore, as output power increases, this bionic cylindrical structure induces greater velocity deficit and higher turbulence intensity behind the rotor. These results provide valuable insights for optimizing aerodynamic designs of wind turbines towards achieving enhanced efficiency for converting wind energy.

## 1. Introduction

As a form of renewable energy, wind power offers infinite accessibility, widespread distribution, environmental benefits, and low electricity costs, making it one of the most promising technologies in the field. In recent days, horizontal-axis wind turbines used to generate wind energy have been divided into upwind and downwind turbines. Pre-bending and coning methods should be adopted in blade design for upwind forms to avoid blade collision with wind turbine towers during operation [1,2]. While the design of a downwind rotor does not necessitate consideration of blade–tower collision, it allows for lower blade stiffness compared to an upwind rotor. By reasonably designing the cone angle and stiffness, the load in the direction of the brandishing can be reduced, and the blades can be more flexible [3]. Loth et al. [4] conducted a numerical simulation of a 13.2 MW downwind wind turbine under steady-state conditions. They showed that the double-blade design of a megawatt downwind mold could save 25% of the mass of the wind turbine and reduce the average blade loading at all operating speeds. In addition, the damage-equivalent loads on the blades are reduced by 60% under stable wind conditions. Frau et al. [5] conducted numerical simulations to compare the output power of upwind and downwind multi-megawatt wind turbines. Compared with the upwind case, the downwind output power was 3% higher, the thrust was 3% higher, and the unsteady load between peaks was three times exceeded due to the higher axial speed on the blade. Zalkind et al. [6] used the FAST aeroelastic program to optimize an upwind three-blade wind turbine into a slimmer two-blade downwind turbine. A 13 W wind turbine with a blade length increased by 25 m was designed. The simulation results indicated that the optimized wind turbine exhibits an 11% increase in annual wind energy yield, a 10% reduction in peak blade load per 5° cone angle, and a decrease in total blade mass.

However, when the inflow passes through the downwind rotor, due to the blocking effect of the tower, the rotating blades will produce an apparent pulsating aerodynamic load, which is called the tower shadow effect. This could cause structural vibration and aerodynamic noise and reduce the fatigue life and output power. Regarding aerodynamic research on downwind rotors, Reiso and Muskulus [7] analyzed the two-dimensional flow field behind cylindrical and truss towers. They concluded that the primary contributors to blade fatigue load were average velocity deficit and turbulence. In contrast, for cylindrical towers, unsteady wake may cause the blade fatigue load to increase by approximately 3%. Sun et al. [8] devised simulation calculations for 10 MW wind turbines with different cone angles and revealed that upwind wind turbines had more output power, smaller thrust, wake deficit, and affected areas than downwind wind turbines. Moreover, downwind wind turbines performed worse than upwind cases in reducing blade root bending moment at large velocities. Therefore, for the past few years, a multitude of scholars have been engaged in investigating strategies to optimize the aerodynamic characteristics of downwind wind turbines with the aim of minimizing safety hazards and enhancing output power [9,10,11].

Kress et al. [12,13] investigated the aerodynamic characteristics of an unsteady-state rotor in a downwind form. They discovered that downwind wind turbines were easier to yaw control and generated more power (approximately 5%). The effects of tilting and coning on a downwind rotor were the opposite of those on an upwind rotor. With increasing tilt angle, the fluctuation of the downwind turbine decreases. Increasing the cone angle could increase the torque and thrust fluctuations simultaneously. However, from a design point of view, a moderate cone angle was desirable for downwind turbines because including a cone in the design enhanced the yaw stability. Meng et al. [14] designed a downwind rotor with forward-folded blades. Through wind tunnel tests, they proved that the power performance of the designed wind turbine remained relatively high compared with the original rotor. By adjusting the blade pitch and cone angle, constant power could be maintained. In addition, by implementing blade-forward folding techniques, the peak bending moment of the blade root can be reduced by 24.1%. Wang et al. [15] conducted comparative research on the aerodynamic performance of downwind and upwind wind turbines utilizing a wind tunnel test and discovered that under identical inflow conditions, the output power of a downwind wind turbine was reduced by 3.2% compared with that of an upwind form. Moreover, particle image velocimetry measurements found that the deficit under the lower half wake of an upwind wind turbine was greater than that of the downwind case. However, the conclusion about power characteristics obtained was inconsistent with that reached by Kress et al. [12]. A possible reason for the contradiction might be that the test conclusion was affected by the electromechanical conversion efficiency of different generators [16]. In addition, field tests were carried out to quantify the influence of tower shadows on unsteady-state downwind forms. Simpson et al. [17] used field test data and OpenFAST software (AeroDyn V15) to evaluate and model the tower shadow effect of downwind wind turbines. The results verify that a downwind wind turbine was feasible, and the tower shadow effect was relatively small compared with turbulence and wind shear. However, the fatigue caused by tower shadows must also be analyzed and considered in the design.

On the other hand, a number of scholars have begun to apply bionics to the design of engineering applications. The bionic design focuses on the effective integration of structure and function, and the purpose is to make the design object have the functions of similar structures [18,19,20]. Through studying the vibrissae of harbor seals, compared with cylinders, the scholars found that their particular three-dimensional structures uniquely impact their vibration response and the flow characteristics of the surrounding flow field, which plays a remarkable flow control in suppressing vortex-induced vibration and reducing lift drag fluctuation. When the inflow passes through the cylinder, the alternating vortices periodically fall off the surface of the object to produce pulsating lift in the lateral direction and drag in the streamwise direction. In contrast, the bionic cylinder formed a transition zone, which could significantly reduce the drag and the pulsation in the near-wake region [21,22]. Wang and Liu [23] compared experimental studies on several related cylinder models. They revealed that the pulsation in the wake of such bionic cylinder structures was apparently attenuated through the turbulence characteristics and spatial and temporal cross-correlation analysis. Moreover, the Karman vortex street in the nodal and saddle planes behind the bionic cylinder drops off. Compared to the other designed models, the wake flow of the bionic cylinder exhibited a weak correlation and greater stability. Based on a vibrissa-shaped cylinder, Chen et al. [24] designed three bionic cylinder models with different wavelengths and adopted wind tunnel experiments at a Reynolds number (Re) ≈ 50,000. The results indicate that the bionic configuration can effectively reduce the average drag and mitigate lift fluctuations to a certain extent. In addition, the bionic structure can disrupt the coherence of near-wake flow around the test model, leading to reduced correlation and turbulent kinetic energy in the wake, as well as decreased average drag and pulsating lift of the experimental models. The optimum results in the model were obtained when the angle of attack was 0°; 15% drag reduction and 58% maximum pulsation lift suppression were achieved. Song et al. [25] studied the single-degree-of-freedom fluid-induced vibration characteristics of a single whisker model and whisker model array in a uniform flow and wake flow in a water tank. They analyzed the correlation between the model vibration response and vortex movement. The findings indicated that this structure has remarkable drag reduction and vibration suppression. Bunjevac et al. [26] investigated the wake characteristics of both a realistic seal vibrissa cylinder and a smooth whisker cylinder at Re = 110 and 390. When the long axis of the shape cross-section was parallel to the incoming flow, the reverse flow region behind the wavy whiskers was reduced, and the turbulence intensity was significantly inhibited. When the angle of attack was disregarded, the power spectral density of the bionic cylinder wake exhibited a reduction of approximately 40% compared to that of the circular case, which indicated that the bionic cylinder has better hydrodynamic performance. In terms of the numerical method, Chu et al. [27] studied the vortex-shedding mode of the wake flow of seal vibrissae at a high Re ≈ 20,000. The data in this research suggest that the wake had unique three-dimensional characteristics, and the average drag and lift coefficient fluctuations decreased by 59.5% and 87.7% compared with the cylinder, respectively. Yoon et al. [28,29] used numerical simulation to perform comparative studies between a smooth cylinder and a helically twisted elliptic cylinder. They found that the fluctuations in drag and lift of the elliptical cylinder were significantly smaller than those of the circular cylinder. The Strouhal number (St) of the elliptic cylinder was reduced by approximately 4% to 7% compared with that of the smooth cylinder, and the smaller the St was, the longer the vortex length was.

In summary, this three-dimensional structure has a high application value for research on the aerodynamic optimization design of wind turbines. However, from the references mentioned above, it can be stated that this bionic cylinder structure has yet to be incorporated into wind turbine design [30], and several challenges remain unresolved. For instance, most current studies focus on the aerodynamic force of a single seal vibrissa cylinder, and more designs need to consider the practical application in wind power engineering. Because of the unique flow field structure behind the seal vibrissa cylinder, it seems that the flow field behind the downwind wind turbine tower, which is significantly affected by the tower shadow interference, can be improved, and the periodic vortex shedding can be suppressed to reducing drag and to achieve power improvement. Therefore, this paper aims to analyze the influence of bionic towers on the aerodynamic performances and wake characteristics of downwind wind turbines. Hence, this study employed an experimental approach to investigate the impact of wind turbine aerodynamic characteristics by utilizing a bionic tower design. Cylindrical and bionic towers were adopted to conduct power and wake comparison tests of a downwind model wind turbine and discuss the results. These results can be used to verify the feasibility of applying downwind bionic tower wind turbines, thereby increasing the possible wind energy conversion efficiency and reducing costs.

The paper is structured as follows: The second section provides a description of the bionic cylinder model, and the third section introduces the experimental setup, including details on the wind turbine model and measurement settings. Section 4 compares and analyzes the flow field characteristics behind a single-cylinder model with the power and wake characteristics of the downwind wind turbine. Finally, in the last section, we present our experimental conclusions.

## 2. Design of the Bionic Tower

**Figure 1:**
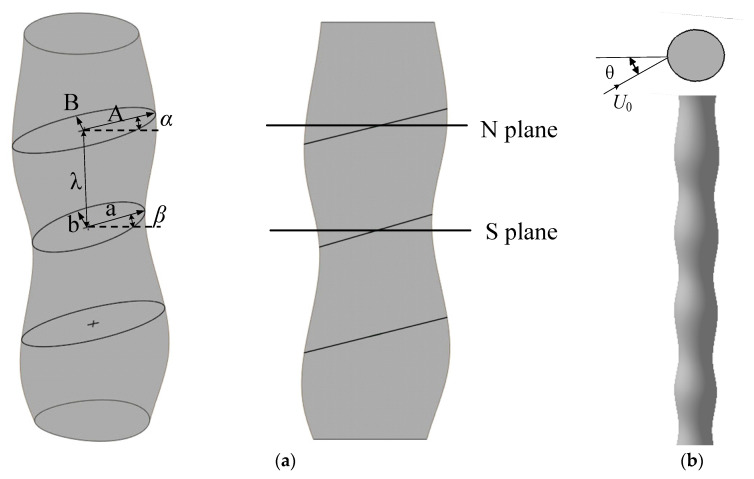
Dimensions of two cylindrical configurations. (**a**) Vibrissa-shaped cylinder; (**b**) bionic cylinder described in this paper.

After extensive analyses of seal vibrissa, the wave-shaped model has been sorted out [23,31]. The shape of seal vibrissae has three characteristics: a wavy surface, an elliptical horizontal section, and an inclined angle of the section. These characteristics were all considered in the design of wind turbine towers. The horizontal section shape of the seal vibrissa is similar to an ellipse, and the size of the elliptical section is different at each horizontal height, leading to a periodic wavy shape. The schematic diagram of the model is shown in Figure 1a. Two elliptical control sections with different radial sizes appear alternately along the axial direction with the period of height λ. These two elliptical control sections have inclination angles α and β with the horizontal plane, resulting in periodic alternating saddle and nodal planes along the height direction. The nodal plane, denoted as the N-plane, is defined as the most raised portion along the axial direction. Correspondingly, the saddle plane, referred to as the S-plane, represents the most concave part along the axial direction.

Due to the relatively flat shape of the actual harbor seal vibrissa, the implementation of this structure in actual wind turbines might result in strengthened sensitivity to inflow from various directions. Therefore, the dramatic changes in the inflow direction result in large fluctuations in the lift drag coefficient of the tower, which will lead to poor efficiency of wind energy conversion when the rotor operates under yaw conditions. To address this issue, this wave-shaped model was modified, and the bionic cylinder was expected to improve the aerodynamic characteristics and adapt to different attack angles. The introduction of an aspect ratio (AR) concept allowed for quantifying the flatness of the control section of the seal vibrissa model. The AR can be calculated as the ratio of the long and short axes of the elliptic control section projected on a horizontal plane perpendicular to the center axis, as shown in Equation (1).

(1) $$\mathrm{AR}=\frac{A\cos\alpha }{B}=\frac{\mathrm{acos}\beta }{b}$$

where A and B are the lengths of the long and short semiaxes of the first elliptical control section, respectively. Similarly, the second elliptical control section is characterized by lengths a and b, denoting the long and short semiaxes, respectively. In addition, *α* and *β* represent the angles between each of these control sections and the horizontal plane, respectively.

According to Equation (1), the aspect ratio of the actual model is approximately 2.38 to 1.54. Suppose the wind turbine is expected to have preferable output power under yaw conditions. In that case, the sensitivity of the bionic cylinder model to the angle of attack should be low. It requires the described aspect ratio to be designed close to 1.0, which means the cylinder is closer to cylindrical. Therefore, we developed a bionic tower model with a constant aspect ratio of 1.2, as shown in Figure 1b. It should be noted that AR = 1.2 is not necessarily the optimum design of the model but only the preliminary proof of the feasibility of the bionic tower design of a downwind wind turbine. The diameter of the actual harbor seal vibration was only approximately 1 mm. To facilitate the model wind turbine design, according to the configuration from Wang and Liu [23,31], the diameter of the designed model was enlarged to 20 mm during this test. Namely, the diameter D_h_ of the bionic cylinder was 20 mm. Table 1 provides the specific parameters of the modified model. Meanwhile, a circular cylinder with the same diameter (i.e., 20 mm) was selected for subsequent comparative research. In the following discussions, the N-plane and S-plane are the typical positions of the bionic cylinder used to investigate the wake characteristics, and this wave-shaped cylinder is referred to as the bionic case.

## 3. Experimental Setup and Methodology

This experiment was conducted in the wind tunnel laboratory at Yangzhou University, which features a closed low-speed wind tunnel with two test sections and an entire steel structure. The two test sections were arranged in series, and the experiment was conducted in the low-speed test section with dimensions of 3.0 m (length), 3.0 m (width), and 3.0 m (height). In addition, the inflow velocity adjustment range spanned from 0 to 25 m/s. A third party independently calibrated the flow field of the wind tunnel, and the quality reached the Chinese national standard GJB1179A-2012 [32]. More detailed information about this wind tunnel can be found in Refs. [33,34].

### 3.1. Wake Measurement of a Single-Cylinder Model

**Figure 2:**
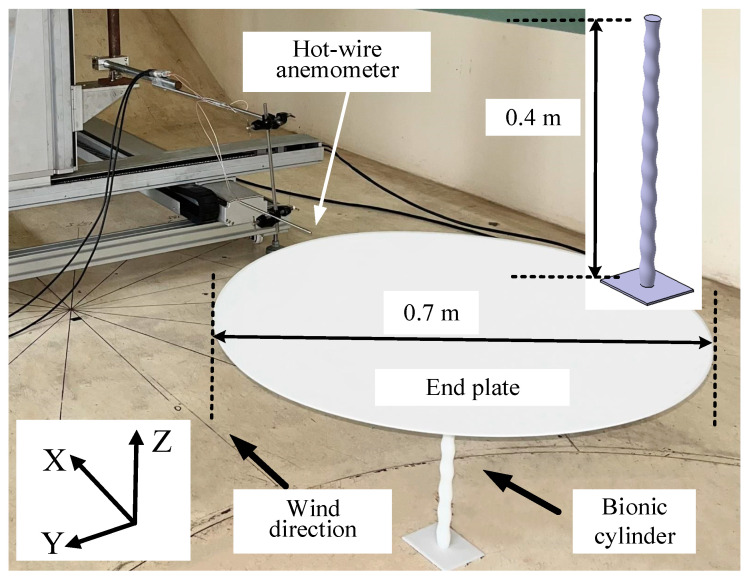
Schematic of the experimental setup.

During the wake measurement test of a single-cylinder, modeling was carried out according to the parameters in Table 1. An end plate with a diameter of 0.7 m and a thickness of 0.005 m was added to the top to suppress the end effect, and a chassis with a length, width, and height of 0.1 m × 0.08 m × 0.003 m was added to the bottom of the cylinder. All models were 3D printed with resin materials, as shown in Figure 2. X, Y, and Z in the coordinate system represent the streamwise (*u*), lateral (*v*), and vertical (*w*) velocity directions, respectively.

The experiments were conducted under uniform inflow conditions with an inflow velocity of 8 m/s. The diameter of the cylinder was taken as the characteristic length, resulting in the Re of approximately 1.1 × 10^4^. In the single-cylinder experiment, the direction of the inflow was parallel to the projection direction of the long semiaxis of the elliptic control section of the cylinder. The wake was measured by a hot-wire anemometer and a two-dimensional probe (55P61, Dantec Dynamics A/S, Copenhagen, Denmark). The hot-wire probe was placed behind the model to reduce possible flow interference. A motorized moving frame (WNMC400, Winner Optical Instruments, Beijing, China) was employed to ensure accurate measurement of the hot wire. In this paper, the sampling frequency of the hot wire was 5 kHz, and 100,000 data points were collected at each measuring point. This was because multiple repeated samples can overcome the random uncertainty in the experiment, thus reducing the influence of uncertainty [35,36].

**Figure 3:**
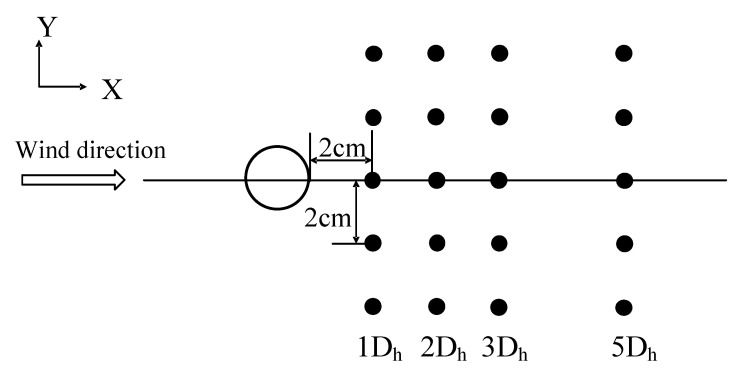
Schematic diagram of the measurement layout behind the cylinder.

The measurement points are shown in Figure 3. These points were at the center height position of the cylinder. In contrast, the bionic cylinder measured the position of the N and S planes, with 20 points in each case. Four columns were set at the positions of 1D_h_, 2D_h_, 3D_h_, and 5D_h_ along the positive direction of the *X*-axis, and the interval between the measuring points was 1D_h_. The turbulence intensities in streamwise direction *I_u_* and lateral direction *I_v_* are calculated as σ*_u_*/*U* and σ*_v_*/*U*, respectively, where σ*_u_* and σ*_v_* are the root mean square of the local average velocity *U*. In addition, according to the results of our previous tests [37], when the height of the bottom wall of the wind tunnel exceeded 0.1 m, the average velocity and turbulence intensity of the inflow basically remained constant with increasing height. The height of the measuring point involved was approximately 0.2 m, so the effect of the boundary layer could be ignored.

### 3.2. Downwind wind Turbine Measurement

**Figure 4:**
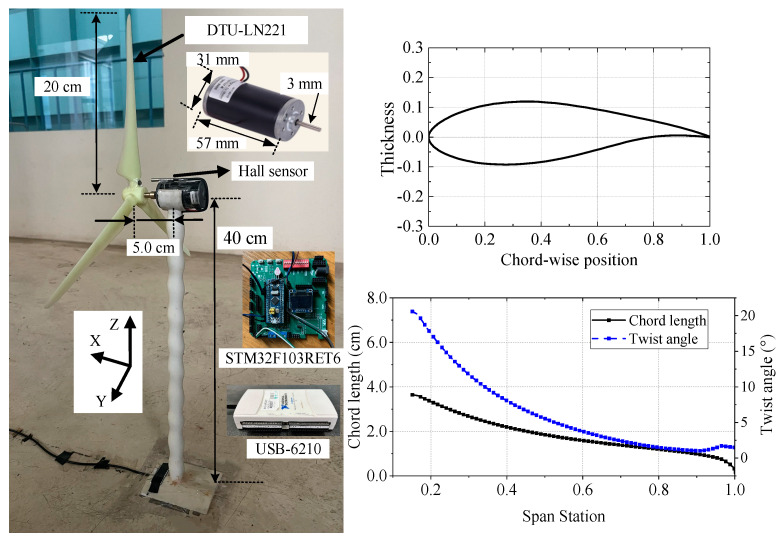
Arrangement of the model turbine and the parameters.

The model turbine, depicted in Figure 4, had a tower height of approximately 0.4 m, a rotor diameter of 0.4 m, and a distance of 5 cm between the rotor and the tower. The base was glued to the bottom wall of the wind tunnel, and the top of the tower was designed with a groove, which was convenient for placing the miniature DC motor (31ZYT57-R, Weisheng Co., Ltd., Dongwan, China). The motor was connected in series with the sampling resistor and adjustable load, and the rotational speed can be changed by adjusting the load resistance. A hall sensor was placed on the upper part of the motor, and the sensor outputs the switching value to the microprocessor (STM32F103RET6, Texas Instruments, Dallas, TX, USA) to accurately measure the rotational speed through the above method. The data collector (USB-6210, National Instruments, Austin, TX, USA) was connected in parallel at both ends of the sampling resistor, converting the collected voltage into the current to calculate the wind turbine’s output shaft power. The rotor blades were all composed of DTU-LN221 airfoil. The Technical University of Denmark developed this airfoil with a relative thickness of 21%. This wind turbine airfoil has been verified in several studies for its aerodynamic characteristics [34,38], and its geometry is shown in the upper right of Figure 4.

The MIRAS method, an interactive rotor aerodynamic simulation method, was utilized for the test rotor [39]. Aerodynamic optimization of blade root and tip was conducted considering the lower order of magnitude of Re. In addition, mirror processing was performed on the original wind turbine to account for downwind operation. The chord length and twist angle distribution are presented in the lower right of Figure 4.

The indirect method is adopted for this experiment [40]. A dynamic torque meter and the data collector were used to obtain data on the tested motor’s shaft power, corresponding rotational speeds, and output currents under different working conditions. The collected data above were fit to bring a functional relationship between the output shaft power, rotational speed, and output current. During actual measurement, the rotational speed of the rotor and output current were collected; thereby, the output shaft power of the model wind turbine could be calculated. Huang et al. [41] conducted repetitive experiments and numerical calculations to compare the power coefficients, verifying the accuracy of this method.

As is well known, the primary focus when optimizing a wind turbine should be on the output power. To analyze the power characteristics, the time-averaged power coefficient C_p_ is defined as Equation (2),

(2) $$C_{p}=\frac{P_{\mathrm{mech}}}{0.5\rho U_{0}^{3}\pi R^{2}}$$

where *R* is the blade radius, *P*_mech_ is the shaft power, which is calculated from the mentioned method above, *U*_0_ is the inflow velocity, and *ρ* is the air density. The maximum blocking ratio corresponding to the swept area of the rotor was approximately 1.40%, so the effect of wind tunnel blocking could be ignored, and the diffusion area could be fully developed during the wake test.

The sampling frequency of the data collector was set at 10 kHz. That is, the time interval was 0.1 ms. The maximum rotational speed of the wind turbine was approximately 2200 RPM. Therefore, the sampling frequency at this time was much higher than the rotating frequency. In addition, the sampling time was set to 5.0 s, with a total of 50,000 sampling data points, ensuring that each condition collected at least 50 rotation cycles. When analyzing the power characteristics, the inflow velocity was also 8 m/s, in keeping with the previous single-cylinder test to ensure the tip speed ratio was adjusted to match the tip speed ratio of a full-scale wind turbine as possible (i.e., kinematic similarity). Using the wind turbine diameter as the characteristic length, the corresponding Re was approximately 2.2 × 10^5^. The power coefficients of the downwind wind turbine of the cylindrical and bionic towers were measured under yaw angles of −15°, −7.5°, 0°, 7.5°, and 15°. The definition of yaw angle θ is shown in Figure 1b, which means that the yaw angle was positive in the clockwise direction of rotation.

**Figure 5:**
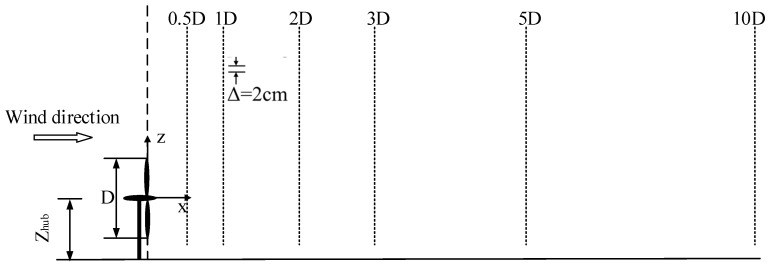
Schematic diagram of the coordinate system definition downwind of a wind turbine.

The investigation of wake effects is equally important for downwind wind turbines. In the comparison of wake characteristics, the hot-wire configuration agreed with the description in Section 3.1. The velocity was calibrated through a pitot tube during the measurement process, and the internal temperature of the wind tunnel was as consistent as possible to reduce the deviation caused by temperature changes. In addition, the arrangement is shown in Figure 5, with the center of the rotor as the origin. Six measurement positions X = 0.5D, 1D, 2D, 3D, 5D, and 10D were arranged vertically behind the rotor, where D represents the diameter of the wind turbine. Behind the vertical centerline of the model, the height range of each position was Z = −0.3 m to 0.3 m, with a spacing of 2 cm between the points and a total of 31 points in each position. In addition, the test points of the bionic case were identical to those of the cylindrical tower wind turbine. It is worth noting that the scale effect between the model wind turbine and the actual wind turbine, which leads to the thrust coefficient of the model wind turbine, is much lower than that of the full-scale wind turbine. Therefore, similarities need to be considered when conducting wind turbine tests in wind tunnels. The rotor used in this study was developed by Sessarego et al. [39] and was scaled 3.75 times by the prototype wind turbine. During the test, the tip speed ratio of the model rotor was close to the full-size rotor. Therefore, the requirements of geometric similarity and kinematic similarity are basically satisfied. For dynamic similarity, the wind tunnel tests cannot match the large Reynolds number of full-scale wind turbines in nature. Combined with the existing studies on the Reynolds number effect caused by wind turbine scaling [42], there is a certain approximate relationship between the power coefficients when the Reynolds number is 2.2 × 10^4^ to 1.65 × 10^5^. In addition, the wake characteristics in the wind tunnel test might differ from those of the full-size wind turbine. However, based on previous findings [43], turbulence statistics in the wake tend to be similar as the Reynolds number exceeds 9.3 × 10^4^ when taking the turbine diameter as the characteristic length. These requirements were met in this test. As a result, the above studies suggest that experiments with micro-scale turbines are also helpful in obtaining relevant insights into the performance and wake of a turbine. In addition, the results could validate the feasibility of installing bionic cylinders into small-scale wind turbines and provide experimental data to support numerical simulations.

## 4. Results and Discussion

### 4.1. Wake Measurement of a Single-Cylinder Model

**Figure 6:**
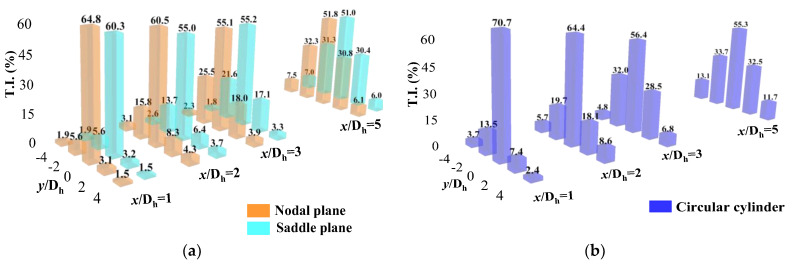
Turbulence intensity (T.I.) descriptions in the wake (streamwise velocity). (**a**) Bionic cylinder; (**b**) circular cylinder.

Figure 6 shows the streamwise turbulence intensity distributions of the two cylinders. The turbulence intensity behind both cylinders in the wake region presents a similar distribution. Furthermore, the turbulence intensity at the circular cylinder’s 1D_h_, 2D_h_, 3D_h_, and 5D_h_ positions was greater than that of the bionic cylinder. The maximum values at these four positions of the circular cylinder were 70.7%, 64.4%, 56.4%, and 55.3%, while the values corresponding to the bionic cylinder were 64.8%, 60.5%, 55.2%, and 51.8%, respectively. This indicated that the motion and energy exchange between vortices at various scales in the wake region of a circular cylinder were more intense than those of a bionic cylinder. Moreover, the energy exchange of fluid micelles on the N-plane of the bionic cylinder was more intense than that on the S-plane. As the distance of the wake flow direction increased, the turbulence intensity behind the cylinder gradually decreased.

**Figure 7:**
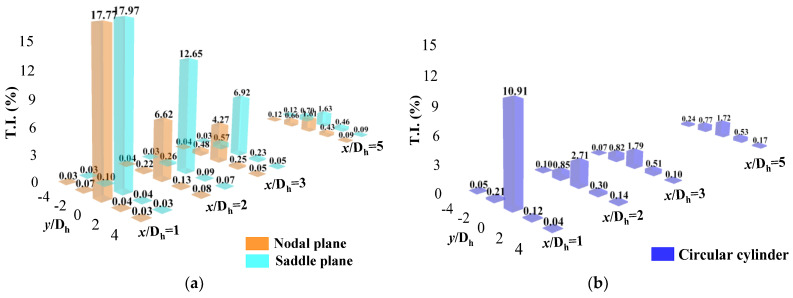
Turbulence intensity descriptions in the wake (lateral velocity). (**a**) Bionic cylinder; (**b**) circular cylinder.

The turbulence intensity of the lateral velocity is also compared in Figure 7. The distribution decreased with increasing flow direction distance. However, the values were much smaller than those in the streamwise direction, and the turbulence intensities at the downstream locations also presented a similar distribution. The maximum values of the circular cylinder at the four downstream locations were 10.91%, 2.71%, 1.79%, and 1.72%, respectively, while the maximum values of the bionic cylinder were 17.97%, 12.65%, 6.92%, and 1.63%, respectively. In contrast, the distribution of the turbulence intensity of the lateral velocity was opposite to that in the streamwise direction. At 1D_h_, 2D_h_, and 3D_h_, the movement of vortices around the bionic cylinder and the energy exchange with the outside were more intense than those of the circular cylinder. In addition, the energy exchange of the bionic cylinder in the S-plane was more intense than that in the N-plane.

**Figure 8:**
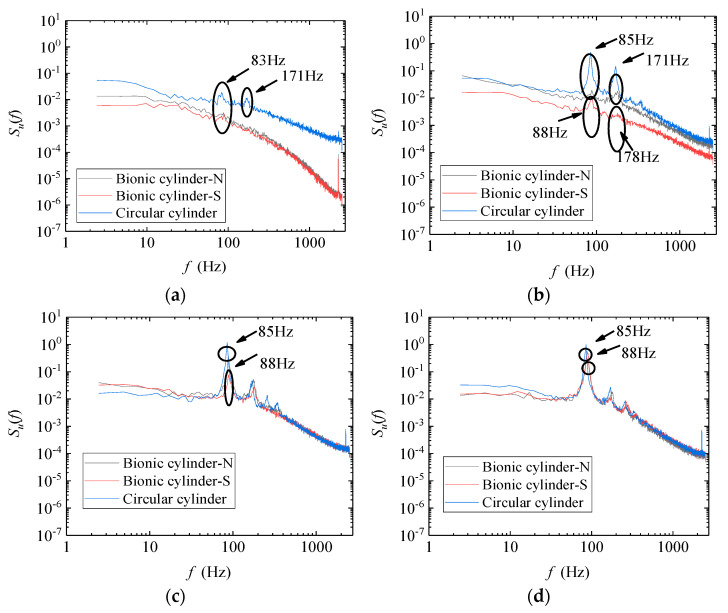
Power spectral density distribution in the center plane of the wake (Y = 0, streamwise velocity). (**a**) X = 1D_h_; (**b**) X = 2D_h_; (**c**) X = 3D_h_; (**d**) X = 5D_h_.

The power spectral density can be used to explain the distribution of fluctuating energy structures at different positions in the wake. Figure 8 compares the power spectral density distribution in the center plane of the wake at several positions directly behind the cylinder. The figure illustrates that the low-frequency pulsation of the two cylinders at the 1D_h_ position contributes prominently to the total pulsation energy, indicating that large-scale vortices behind the cylinder dominate the turbulence disturbance. The circular cylinder exhibited two peak signals broadly higher than the bionic case at approximately 83 Hz (St ≈ 0.2) and 171 Hz (St ≈ 0.4), indicating that the energy corresponding to the pulsation frequency behind the circular cylinder was larger. This means that the movement of vortices behind the circular cylinder was more intense than that behind the bionic cylinder. At the 2D_h_ position of the wake, the circular cylinder case exhibits two peak signals significantly higher than those of the bionic cylinder at 85 Hz and 171 Hz. The turbulence disturbance caused by vortices of different scales behind the circular cylinder was greater, and the disturbance behind the N-plane of the bionic cylinder was more prominent than that at the S-plane. At the 3D_h_ and 5D_h_ positions, the maximum peak signal of the circular cylinder appeared at 85 Hz, while the bionic case appeared at 88 Hz. In addition to the low-frequency domain, the pulsation energy in the wake of both cases gradually tended to be approached. This was because as the distance between the wake regions increased, vortices of different scales continuously collided and dissipated with each other during their movement, decreasing the frequency difference. In summary, the distribution verified that the bionic cylinder could effectively suppress vortex-induced vibration.

**Figure 9:**
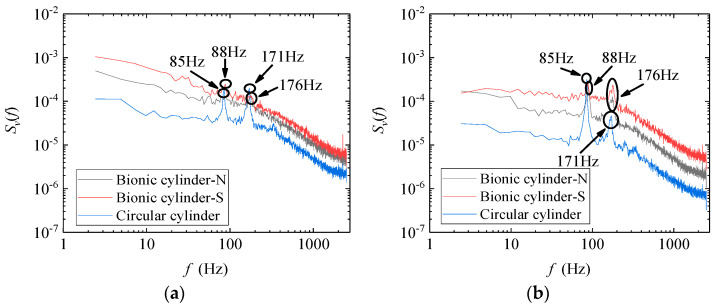
Power spectral density distribution in the center plane of the wake (Y = 0, lateral velocity). (**a**) X = 2D_h_; (**b**) X = 3D_h_.

Due to the three-dimensional flow of the wake, there were also significant differences in the distribution of pulsating energy in the lateral directions. The power spectral density of the lateral velocity at the 2D_h_ and 3D_h_ positions was compared, as shown in Figure 9. The two peak signals of the circular cylinder appear at 85 Hz and 171 Hz. The maximum peak signal of the bionic cylinder appears at 88 Hz and 176 Hz, and the pulsating energy of the circular cylinder was lower than that of the bionic cylinder, which indicates that the turbulence disturbance caused by vortices behind the circular cylinder was smaller than that of the bionic cylinder, consistent with the turbulence intensity results. The pulsating energy behind the S-plane of the bionic cylinder was larger than that of the N-plane, which was opposite to the consequence in the streamwise direction.

### 4.2. Power Characteristics

**Figure 10:**
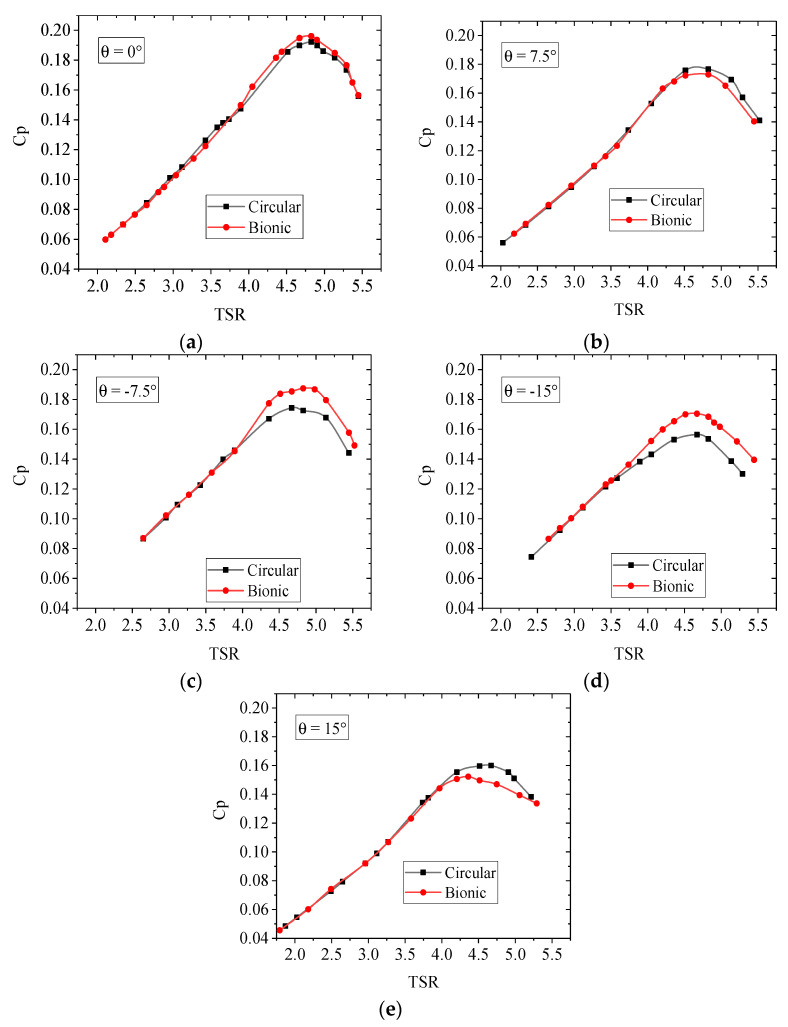
Comparisons of the wind turbine power coefficient between the circular and bionic towers. (**a**) θ = 0°; (**b**) θ = 7.5°; (**c**) θ = −7.5°; (**d**) θ = −15°; (**e**) θ = 15°.

The power coefficient of wind turbines as a function of the tip speed ratio (TSR = *ωR/U*_0_, where *ω* is the angular velocity of the model turbine) under 0° yaw conditions is depicted in Figure 10a. It can be observed that the power coefficients exhibit an initial increase followed by a subsequent decrease with the rise in tip speed ratio. The optimum tip speed ratio under the tested inflow velocity was approximately 4.8. At low tip speed ratios, the change rules of the power coefficient in cylindrical and bionic cases were similar. While at tested tip speed ratios, the maximum power coefficient of the bionic case was 0.196, which had a 2.1% increase over that of the cylindrical. Bastankhah and Porte-Agel [40] point out that the power coefficient of the model wind turbine varies with the tip speed ratio and the inflow velocity. Due to the Reynolds number dependency, a slower flow results in a lower power coefficient. However, the current result agrees with our previous experiment [41] and is relatively close to another observation of Dou et al. [44].

Meanwhile, due to the long-term yaw condition of wind turbines in practice, comparing the power characteristics of wind turbines under the yaw state has essential research value. Hence, the power characteristics of wind turbines with cylindrical and bionic towers under yaw conditions are illustrated in Figure 10b–e. In these figures, the yaw angle is defined as positive when rotating clockwise. As the yaw angle increases, the projected area of the rotor plane in the inflow direction decreases, resulting in a reduction in wind energy flowing into the wind turbine and subsequently leading to a decrease in its output power. Under the conditions of θ = 7.5°, −7.5°, 15°, and −15°, the maximum power coefficient decreased by 4.6%, 11.7%, 13.3%, and 23.5%, respectively, compared to the unyawed case.

At high tip speed ratios, the power coefficient of the bionic case during counterclockwise yaw was greater than that of the cylindrical tower wind turbine. In contrast, the opposite trend occurred during the clockwise yaw condition. This situation may be because the irregular tower structure causes the attack angle of the blade section to change. Compared with the cylindrical case, the bionic model could significantly alter the wake structure. The wake behind the tower experiences noticeable disturbance, leading to variation in the recirculation region and significant attenuation of flow pulsation in the wake [23]. As the inflow angle varies, both the strength and shedding frequency of Karman vortices within the saddle and nodal planes could be notably changed, making a difference in the output power. When the counterclockwise yaw angle was 7.5°, the maximum power coefficient of the bionic case was 7.53% greater than that of the cylindrical tower. When the clockwise yaw angle was 7.5°, the reduced power coefficient was 2.16%. Moreover, when the yaw angle was 15°, the power coefficient increased by 8.98% under counterclockwise conditions and decreased by 4.80% under clockwise conditions. Such a result demonstrated that the increase in the power coefficient of the bionic case under counterclockwise conditions was more evident than the decrease under clockwise conditions. It seems that the bionic tower wind turbine could output more power than the cylindrical tower. In practical applications, the wind turbine could be arranged according to the wind-rose diagram of the average statistics of a region for several years to obtain further wind energy conversion efficiency.

### 4.3. Wind Turbine Wake Characteristics

**Figure 11:**
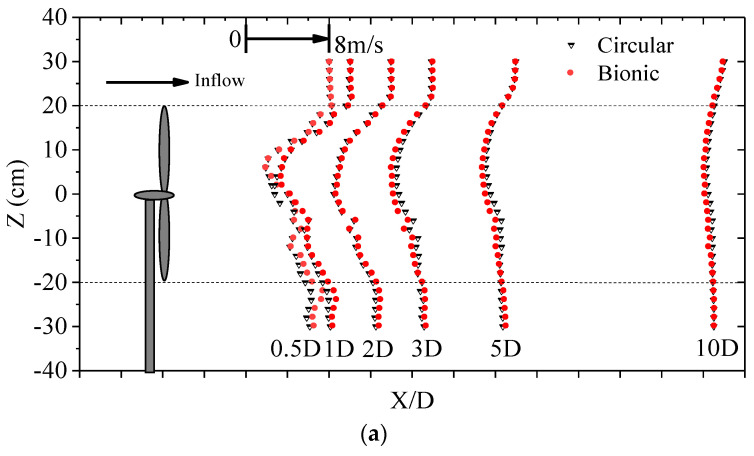

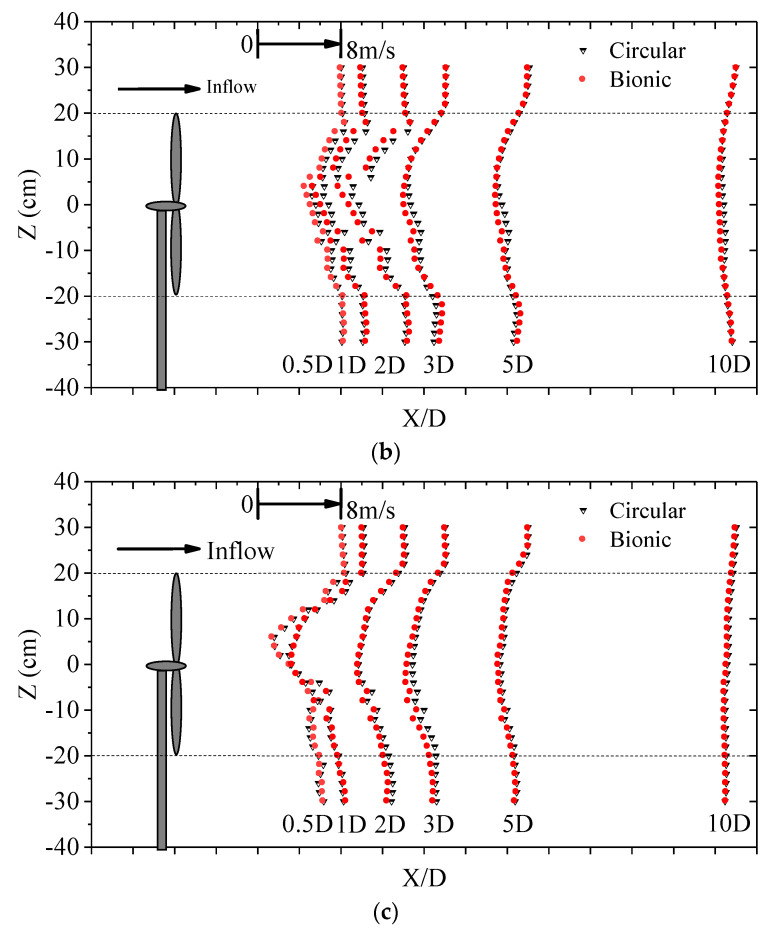
Comparisons of the wake distribution between the cylindrical and bionic tower wind turbines (streamwise velocity). (**a**) θ = 0°; (**b**) θ = −15°; (**c**) θ = 15°.

Choosing θ = −15°, 0°, and 15° as an example, Figure 11a–c illustrate the wake distribution between the two wind turbines. During wake measurements, the tip speed ratio was fixed at 4.7, which closely approximates the optimum position. The presence of a tower causes an asymmetric distribution of wake velocity along its vertical direction. The wake velocities behind the lower tip part are less than those near the upper tip, and the deficit was obvious at Z = −6 to 6 cm behind the center of the wind turbine. At X = 10D, the wake velocities of the two wind turbines overlap, and the main difference appears within the range of X = 5D. Under the condition of θ = 0°, the wake velocity deficit of the bionic case was evidently more minor than that of the cylindrical tower behind the wind turbine in the range of Z = −30 to −20 cm, which indicated that the bionic tower structure had a distinct improvement effect on the wake recovery in this vertical range, and the same result can be found in the counterclockwise 15° yaw condition. In contrast, in the vertical range of approximately Z = −20 to 20 cm behind the wind turbine plane, the velocity deficit of the bionic case was larger than that of the cylindrical case at most measuring positions, especially at X = 3D to 10D. The bionic tower structure could not improve the wake recovery behind the rotor while increasing the power coefficient. It is worth noting that in the yaw condition, the thrust component formed not only in the direction of the inflow but also in the lateral direction, acting on the inflow through the rotor, which could cause different degrees of wake distortion.

For the 15° clockwise yaw condition with a reduced power coefficient, most of the measurement points at X = 0.5D to 5D revealed that the velocity deficit of the bionic case was more severe than that under the cylindrical condition, illustrating that the bionic structure was not conducive to improving the wake recovery at this yaw angle. For all conditions, the increase in the flow direction distance could gradually recover the wake velocity, and the difference in the wake velocity between cylindrical and bionic tower wind turbines became indistinctive. Under 0° yaw conditions, when X = 0.5D, 1D, 2D, 3D, 5D, and 10D, the maximum velocity deficits of the cylindrical tower wind turbine were 76.2%, 90.4%, 69.1%, 44.0%, 36.7%, and 21.6% of the inflow velocity, respectively. In comparison, the bionic tower wind turbines were 76.1%, 82.5%, 66.5%, 49.7%, 40.1%, and 23.0%. The results suggest that the wake deficit of the two model turbines was greater at a 1D position rather than 0.5D closer to the rotor. This may be due to the obstruction of the nacelle; the reverse flow region exists within the range of X = 1D. Meanwhile, owing to the robust development of both the central vortices and tower vortices, there are significant fluctuations in both the vertical and lateral velocity distributions, especially at the position close to the rotation plane. This flow characteristic distribution would have a certain degree of influence on the maximum deficit position of the wake.

By utilizing the blade element momentum (BEM) method, the wake velocity in streamwise and lateral directions could be converted into the axial and tangential induction factors, and the local angle of attack at different cross-sections can be calculated by using the velocities obtained from the two wind turbine cases [45,46,47].

(3) $$\begin{array}{l} \mathrm{AOA}=\arctan\left(\frac{\left(1-a_{1}\right)U_{0}}{\left(1+a_{2}\right)\omega r}\right)-\varphi =\arctan\left(\frac{U_{0}+u}{2\omega r+v}\right)-\varphi \end{array}$$

In Equation (3), AOA is the angle of attack for the turbine blade section at a certain spanwise location; *a*_1_ and *a*_2_ are the axial and tangential induction factors, respectively. *u* and *v* are the streamwise and lateral velocities of the measuring points, respectively. *r* is the spanwise distance from the measuring point to the center of the rotor; *φ* is the twist angle of the corresponding position.

**Figure 12:**
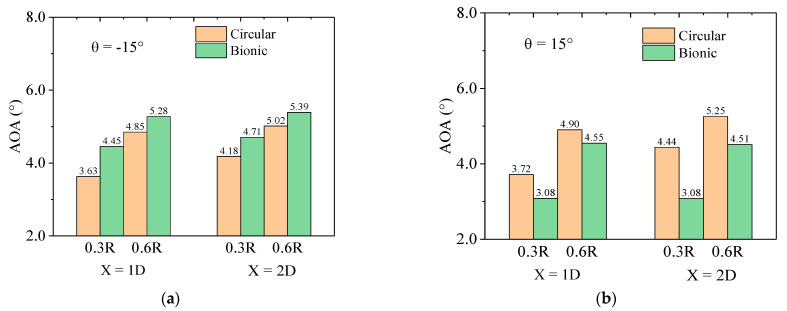
The angle of attack of the cylindrical and bionic case at *r* = 0.3R and 0.6R. (**a**) θ = −15°; (**b**) θ = 15°.

As suggested by Alber et al. [45], *a*_1_ and *a*_2_ elucidate the decline in axial velocity and the augmentation of tangential velocity component from a reference point situated sufficiently distant from the rotor plane, rather than directly adopting the flow velocity passing through the rotor plane itself. The reference point in their study was recorded at a 1.3D position far away from the rotor to reduce the effect of wind tunnel shrinkage. Therefore, the test data of the lower part were selected, and two typical positions of X = 1D, 2D, and *r* = 0.3R and 0.6R were adopted for comparison. The angle of attack at the corresponding positions can be calculated by converting the streamwise and lateral velocities into axial- and tangential-induced velocities. Figure 12 illustrates a comparison of the angle of attack between the bionic and cylindrical tower wind turbines at two positions under the θ = −15° and θ = 15° conditions. The comparisons correspond to the working conditions when the power coefficient increases and decreases remarkably. The discrepancies observed between the results obtained at the two positions can be attributed to variations in blade configurations (i.e., the twist angle), as well as the reduced angular velocity experienced at the 0.3R position.

The findings suggest that the angle of attack at each measurement point on the bionic tower wind turbine exceeds that of its cylindrical counterpart under the θ = −15° case (see Figure 12a). This situation is attributed to the high tip speed ratio in wake measurement, resulting in relatively small angles of attack for both sections and leading them within the linear range of lift coefficient. Consequently, a slight increase could bring the angle of attack of the blade section closer to its optimal value (i.e., a level that approaches the optimum condition of the wind turbine [45]). It can be reasonably inferred that the blade section of the bionic case exhibits a larger lift–drag ratio, which is in accord with the conclusions drawn from the power characteristics in the previous section. As a result, this enhancement contributes to enhancing the power coefficient of the wind turbine and potentially explains the observed disparity between these two turbines. Under the θ = 15° condition (see Figure 12b), the angle of attack of the bionic case was smaller than that of the cylindrical case. The smaller angle of attack reduces the aerodynamic efficiency of the section, which leads to a reduction in power.

**Figure 13:**
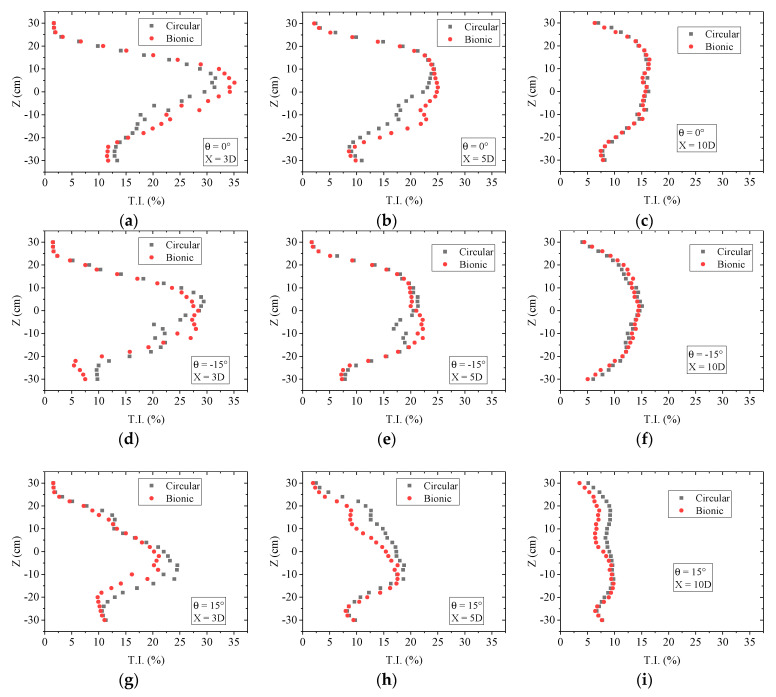
Comparison of turbulence intensity between cylindrical and bionic tower wind turbines (streamwise direction). (**a**) θ = 0°, X = 3D; (**b**) θ = 0°, X = 5D; (**c**) θ = 0°, X = 10D; (**d**) θ = −15°, X = 3D; (**e**) θ = −15°, X = 5D; (**f**) θ = −15°, X = 10D; (**g**) θ = 15°, X = 3D; (**h**) θ = 15°, X = 5D; (**i**) θ = 15°, X = 10D.

The rotation of the wind turbine will cause the movement of vortices behind the rotor and energy exchange with the external flow field so that there will be a certain degree of disturbance in the wake. To reduce interference, the row spacing of wind turbines mentioned in the technical specification for microsite of wind farm engineering in the National Energy Industry (i.e., NB-10103-2018, China [48]) should be more than three times. Therefore, this paper adopts the streamwise wake velocity as an example. Figure 13 shows the distribution of turbulence intensity at X = 3D, 5D, and 10D for cylindrical and bionic tower wind turbines at θ = 0°, −15°, and 15°, respectively. The turbulence intensity was also calculated as σ*_u_*/*U*. The trend of turbulence intensity in the wake region of the two wind turbines along the vertical direction was approximately approached. However, due to the more intense vortex movement and energy exchange, the turbulence intensity could be larger, with the maximum value exceeding 35%. Compared with the conditions of θ = 0° and −15°, the turbulence intensities of the two wind turbines were staggered in the upper part of the wake until the tendency coincided at X = 10D. The difference in turbulence intensity between the two mainly appears in the lower part of the wind turbine. In the vertical range of Z = −30 to −20 cm, the turbulence intensity of the bionic tower case was comparatively smaller than that of the cylindrical tower case in most positions, while the opposite result appeared when Z = −20 to 0 cm. It showed that the bionic tower structure would increase the turbulence intensity directly behind the wake of the rotor while increasing the power. This may be attributed to such wave cylinders having a more evident disturbance effect on the wind turbine flow field. The interaction between the external flow field and the wake region becomes more obvious, and various vortices collide and merge, resulting in more frequent momentum exchange inside and outside the wake region, resulting in an increase in the amplitude of its pulsating energy. In addition, different yaw angles have apparent effects on the turbulence intensity distribution. Under the condition of θ = 15°, the power of the bionic wind turbine decreased, and the turbulence intensity at most wake locations was smaller than that of the cylindrical wind turbine.

## 5. Conclusions

This paper primarily investigates the effect of a bionic tower on the aerodynamic characteristics of downwind wind turbines. It employs testing methods to analyze the wake characteristics between bionic and circular cylinders and conducts comparative studies on the aerodynamic characteristics of wind turbine models with these two towers. The main research findings are as follows:

(1) The single-cylinder results indicate that in the streamwise direction, the movement of vortices in the wake of the circular cylinder is more intense than that of the designed bionic cylinder. In addition, the turbulence intensity in the nodal plane of the bionic cylinder is more intense than that in the saddle plane. In a comparison of the power spectral density, the difference mainly appears in the near-wake position, where the low-frequency pulsation of the two cylinders makes a prominent contribution to the total pulsation energy. Two peak signals of the circular cylinder case appear at approximately 83 to 85 Hz (St ≈ 0.2) and 171 Hz (St ≈ 0.4), which are higher than those for the bionic. That is, the energy corresponding to the pulsation frequency of the circular cylinder is more notable than that of the bionic cylinder. However, for the lateral velocity, the turbulence caused by the vortices of different scales behind the circular cylinder is smaller than that of the bionic cylinder.

(2) The power characteristic test results indicate little difference in the power coefficient and slope between these two wind turbines at low tip speed ratios, while the difference is more significant at high tip speed ratios. The output power of the bionic wind turbine is affected by the inflow angle. Specifically, the bionic tower case exhibits a greater power coefficient under counterclockwise yaw conditions than the cylindrical tower case. However, in clockwise yaw conditions, the result is the opposite. Nevertheless, it is worth noting that the increase in the power coefficient of the bionic tower wind turbine under counterclockwise conditions is greater than the decrease in the power coefficient under clockwise conditions.

(3) The results of the wake characteristics illustrate that while the wind turbine increases the power, the bionic tower structures could cause more velocity deficits directly behind the rotor in a large proportion of wake positions, especially in near-wake regions. By using BEM theory, with the increase in output power, the corresponding angle of attack of the bionic case is larger than that of the cylindrical case, which might make the inflow angle of the blade airfoil section closer to the optimum angle of attack and enhance the power. In addition, the bionic cylinder increases the turbulence intensity directly behind the wake of the rotor while increasing the power.

It should be noted that the test object in this study was a bionic cylinder with an aspect ratio of 1.2, which adopted uniform inflow to preliminarily demonstrate the feasibility of using bionic cylinders in aerodynamic design. However, given the presence of atmospheric boundary layer winds in natural environments, it is clear that differences in flow characteristics will impact the aerodynamic performance of wind turbines. Therefore, future work will involve conducting wind tunnel studies on both bionic cylinder wind turbines with varying AR values and wind shear inflow to reveal superior aerodynamic characteristics for bionic downwind turbines.

## Figures and Tables

**Table 1:** Geometric parameters of bionic cylinder configurations.

	A (mm)	B (mm)	a (mm)	b (mm)	λ (mm)	*α* (°)	*β* (°)	D_h_ (mm)	AR
Bionic cylinder	12.8	10.3	10.2	8.1	18.9	15.27	17.6	20.0	1.2

## Data Availability

The data can be obtained upon request from the corresponding author.
